# Restoring *Ag1*, an ancient regeneration gene lost in amniotes, accelerates skin healing in mice

**DOI:** 10.3389/fcell.2026.1706902

**Published:** 2026-02-19

**Authors:** Anastasiya V. Kosykh, Elena B. Zhigmitova, Nadezhda A. Evtushenko, Tatyana H. Gurskaia, Aleksandra A. Martynova, Yuliya Yu. Silaeva, Anastasiia S. Ivanova, Natalia Y. Martynova, Maria B. Tereshina, Stanislav G. Rudyak, Andrey A. Panteleyev, Helen P. Makarenkova, Nadya G. Gurskaya, Sergey A. Lukyanov, Andrey G. Zaraisky

**Affiliations:** 1 Pirogov Russian National Research Medical University, Moscow, Russia; 2 Institute of Gene Biology, Moscow, Russia; 3 Department of Molecular Medicine, The Scripps Research Institute, La Jolla, CA, United States; 4 Shemyakin-Ovchinnikov Institute of Bioorganic Chemistry, Moscow, Russia; 5 Koltzov Institute of Developmental Biology, Moscow, Russia

**Keywords:** AG1, genes lost in evolution, skin, skin wound healing, tet on expression, transgenic mice, *Xenopus*

## Abstract

Tissue and organ regeneration is a remarkable ability observed in many animal species, which has been significantly reduced or lost in several vertebrate lineages, partly due to the evolutionary loss of regeneration-associated genes. For example, many genes involved in limb and skin regeneration in anamniotes (fish and amphibians) have been lost in amniotes (reptiles, birds, and mammals) following their transition to terrestrial life. This raises the intriguing question of whether reintroducing such lost genes could partially restore regenerative abilities. Here, we investigated whether the ag1 gene, which plays a key role in regeneration in fish and amphibians and was lost in amniotes, could enhance skin wound healing in mice. We generated transgenic mice with inducible expression of the *Xenopus laevis* ag1 gene in the skin and compared wound healing dynamics between induced and non-induced groups. Induction of ag1 resulted in approximately 20% faster wound closure. Transcriptomic analysis revealed enhanced activation of multiple pathways involved in wound repair and, notably, the upregulation of a subset of genes typically associated with scarless healing in amphibians and mammalian fetuses. Altogether, our findings demonstrate that a gene lost more than 300 million years ago can still stimulate reparative processes in mammalian tissues, highlighting the potential of ancient gene reactivation to enhance tissue repair in modern vertebrates.

## Introduction

During vertebrate evolution, many genes involved in appendage regeneration in anamniotes (fish and amphibians) were lost in amniotes (reptiles, birds, and mammals) following their transition to terrestrial life. This loss likely reflected a trade-off for adaptive innovations such as skin keratinization, increased immune surveillance, and larger body size, which improved terrestrial survival but restricted regenerative capacity (Zaraisky et al., 2024). As a result, many regeneration-associated genes were no longer maintained by natural selection and were eventually lost (Alibardi, 2020; Zaraisky et al., 2024). In placental mammals alone, around 150 such regulators have been identified (Lyubetsky et al., 2023; Zaraisky et al., 2024).

Because multiple genes and network components were lost, restoring limb regeneration in mammals by reintroducing single genes is unlikely. Indeed, limb regeneration has not reappeared in any amniote lineage, indicating progressive degradation of the ancestral regenerative network over hundreds of millions of years (see review in Zaraisky et al., 2024).

Some of these lost genes, however, continue to function in other reparative processes in anamniotes that remain conserved in mammals. One such process is skin wound healing. Although modified by terrestrial adaptations, it retains many ancestral features (Abe et al., 2020).

One major difference between skin wound healing in anamniotes and amniotes is the speed of re-epithelialization. In salamanders, wound closure by newly formed epithelium occurs within 10 h, whereas in mice it takes 2–3 days (Tseng and Levin, 2008; Mu et al., 2014; Levesque et al., 2010; Feng et al., 2021). In humans, re-epithelialization of skin wounds proceeds even slower: about twice as long as in mice (Matsumoto-Oda et al., 2025).

Another difference is scarring. Adult amphibians heal without scarring (Yokoyama et al., 2011; Seifert et al., 2012), whereas adult mammals typically form scars (Ferguson and O'Kane, 2004; Gurtner et al., 2008). Remarkably, mammalian fetal wounds heal rapidly and scarlessly, resembling amphibian regeneration (Stocum, 2017; Martin, 1997; Ferguson and O'Kane, 2004; Yokoyama et al., 2011). This regenerative type of skin healing restores tissue architecture to its original state.

Although the molecular basis of this perfect wound healing in adult amphibians healing is not fully understood, several genes implicated in limb regeneration appear to play a role (Kawasumi et al., 2013). Among them is *ag1*, a member of the *Agr* gene family encoding secreted disulfide isomerases. While *agr2* and *agr3* are conserved in all vertebrates, *ag1* was lost in amniotes and persists only in anamniotes (Ivanova et al., 2013). In adult salamanders and pre-metamorphic frog tadpoles, *ag1* and *agr2* are expressed in the wound epithelium and regulate limb regeneration (Kumar and Brockes, 2012; Ivanova et al., 2013; Ivanova et al., 2021). *Agr2* has also been reported to be activated in response to skin wounding in mice and, as in amphibians, it promotes wound re-epithelialization (Zhu et al., 2017; Kosykh et al., 2023).

In this study, we tested whether reintroduction of the lost *ag1* gene from *Xenopus laevis* could enhance skin wound healing in mice. Using a doxycycline-inducible system, we found that *ag1* expression accelerated wound closure by approximately 20% during the early healing phase compared with controls. Transcriptomic analysis of wound tissues revealed activation of multiple wound-healing pathways and upregulation of genes associated with scarless healing, including *Col3a1*, *Col5a2, Twist2*, *Prrx2*, and *Tnc*.

These findings demonstrate that the evolutionarily lost regeneration regulator *ag1* can functionally integrate into mammalian repair mechanisms and enhance wound healing. This supports the broader concept that reintroducing ancestral regeneration genes may offer new strategies to improve tissue repair in mammals, including humans.

## Results

### Effect of recombinant Ag1 on viability, proliferation, and migration of immortalized human keratinocytes

To determine whether frog Ag1 could influence mammalian skin cells, we first examined its effects on the viability, proliferation, and migration of immortalized human keratinocytes (HaCaT) and human dermal fibroblast (hdF) *in vitro*. Addition of recombinant Ag1 protein to HaCaT cultures did not alter cell viability at seeding or during subsequent days, indicating no impact on keratinocyte adhesion (data not shown).

**FIGURE 1:**
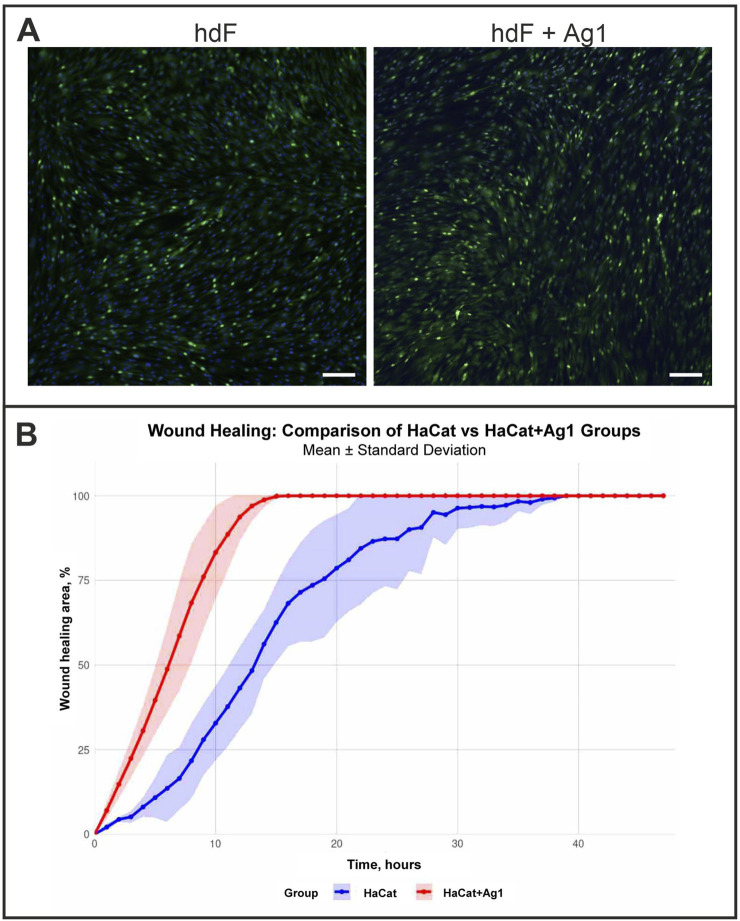
Effects of recombinant Ag1 protein on cell proliferation and migration. **(A)** Representative immunohistochemical staining for Ki67 in human dermal fibroblasts (hdF) cultures grown with or without recombinant Ag1. No significant differences in proliferation were observed between groups. Bar: 50 µm. **(B)** Kinetics of wound closure in HaCaT keratinocyte scratch assays. The graph depicts the percentage of wound healing area over time for control HaCat cells (blue) and HaCat cells treated with Ag1 (HaCaT + Ag1, red). Data are presented as the mean ± standard deviation (shaded area). The HaCaT + Ag1 group exhibited a significantly accelerated rate of wound closure compared to the untreated control.

For migration analysis, confluent HaCaT monolayers were treated with recombinant Ag1 (5 ng/μL) and compared with untreated controls. Immunostaining for Ki67, a proliferation marker, revealed no difference in the proportion of positive cells between groups after 48 h (Figure 1A), suggesting that Ag1 does not influence proliferation.

In contrast, scratch-wound assays showed that Ag1 treatment markedly enhanced keratinocyte migration relative to controls (Figure 1B). Time-lapse video microscopy over 48 h demonstrated accelerated wound closure in Ag1-treated cultures, with the most pronounced effect occurring between 5 and 15 h after scratch induction. We analyzed wound-healing trajectories between control and experimental groups with a linear mixed-effects model to account for repeated measurements of the same samples over time. Critically, type III F test showed that the group-by-time interaction was significant (p = 5.73 × 10^-10), indicating that the pattern of healing over time differed between groups. These results suggest that the experimental treatment altered the trajectory of wound healing relative to control. These findings indicate that Ag1 can promote migration without affecting proliferation in human keratinocytes.

### Generation of a conditional *ag1* expression system

**FIGURE 2:**
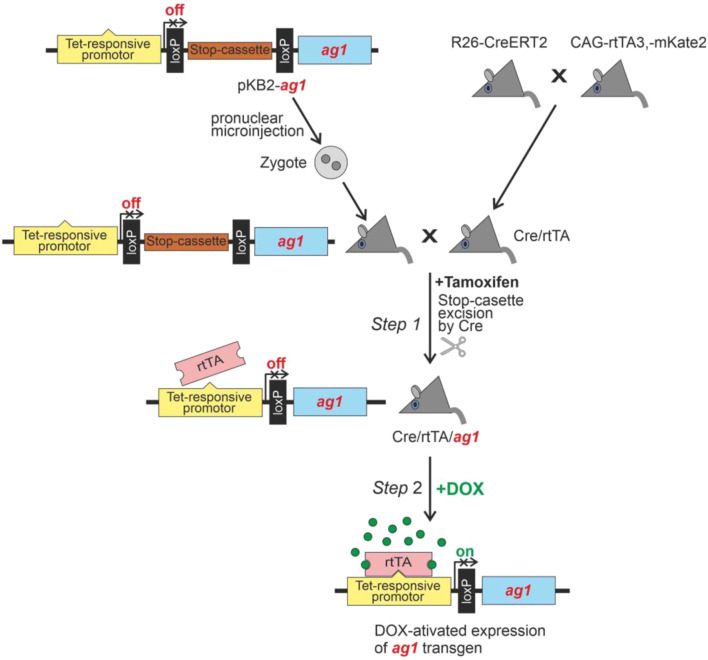
Schematic representation of *Xenopus laevis* ag1 expression induction in transgenic mice.

To investigate the functional consequences of *ag1* expression in mammals, we created a doxycycline-inducible transgenic system based on the Tet-On regulatory cassette, allowing spatiotemporal control of Ag1 production in mice. The *ag1* cDNA *Xenopus laevis* (NM_213699.1) was cloned into the pKB2 plasmid downstream of the Tet-On promoter and upstream of a LoxP-flanked Stop cassette, which blocks transcription until excised by Cre recombinase (Figure 2; see *Materials and Methods* for details). The resulting construct (pKB2-*ag1*) was introduced into the mouse genome by pronuclear injection.

Activation of *ag1* expression *in vivo* occurred in two sequential steps. In *Step 1*, the Stop cassette was removed through Cre-mediated recombination by crossing pKB2-*ag1* mice with double-transgenic animals expressing CreERT2 and rtTA, followed by tamoxifen administration to induce Cre activity (Figure 2, *Step 1*). In *Step 2*, *ag1* transcription was initiated by doxycycline administration, which activates the Tet-On promoter in the presence of the rtTA transactivator (Figure 2, *Step 2*).

### Validation of the inducible *ag1* expression system *in vitro* and *in vivo*

**FIGURE 3:**
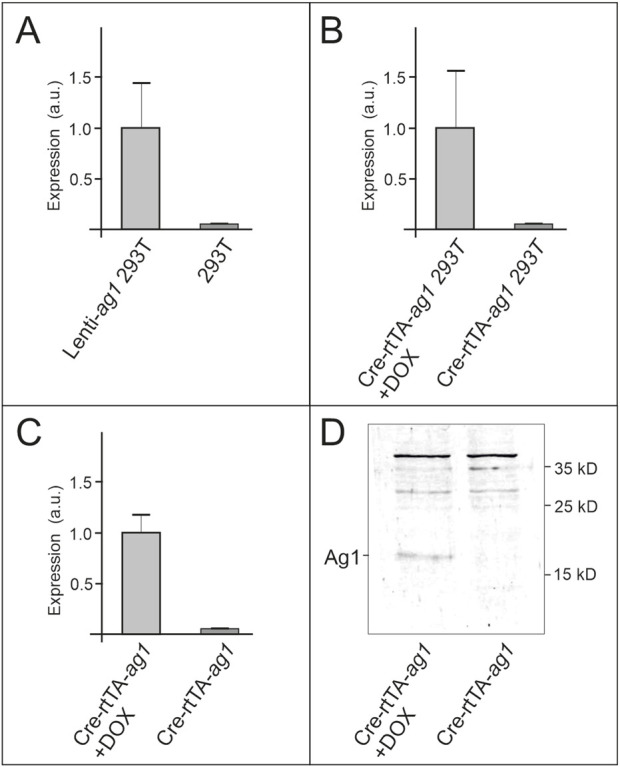
Analysis of *ag1* expression in HEK293T cells and transgenic mice. All data presented as the average of three independent replicates with standard deviations. Statistical significance was assessed by a paired Student’s t-test. **(A)** Real-time quantitative PCR (RT-qPCR) results for *ag1* mRNA levels in HEK293T cells are depicted before and after expression of *ag1*-encoding lentiviral construct. **(B)** RT-qPCR analysis of *ag1* expression in HEK293T cells co-transfected with pCre-BFP, prtTA, and pKB2-ag1 before and after doxycycline induction. **(C)** RT-qPCR measurement of *ag1* mRNA in skin from Cre/rtTA/*ag1* mice prior to and following 7 days of doxycycline administration. **(D)** Western blot analysis of skin lysates from Cre/rtTA/*ag1* mice before and after 7 days of doxycycline treatment, probed with Ag1-specific antibodies.

To evaluate the functionality of the conditional *ag1* expression system, we first tested inducibility in HEK293T cells. Cells were co-transfected with pKB2-*ag1*, pCreBFP, and prtTA plasmids, whereas a stably transduced HEK293T line constitutively expressing Ag1 (Lenti-*ag1*-293T) served as a positive control. qRT-PCR analysis showed that *ag1* transcripts were undetectable in co-transfected cells in the absence of doxycycline, but were robustly induced following a 48 h doxycycline treatment (Figures 3A,B). These results confirm that the pKB2-*ag1* construct is responsive to tetracycline-inducible activation and exhibits minimal background expression.

We next validated system performance *in vivo*. qRT-PCR analysis demonstrated a significant upregulation of *ag1* mRNA in skin samples from Cre/rtTA/*ag1* mice after 7 days of doxycycline administration compared with non-induced controls (Figure 3C). Western blot analysis further confirmed Ag1 protein expression exclusively in doxycycline-treated animals, with a specific ∼18 kDa band detected only in induced samples (Figure 3D). Collectively, these experiments demonstrate that the transgenic system produces tightly controlled and efficient *ag1* expression at both the mRNA and protein levels *in vitro* and *in vivo*.

### Assessment of skin wound healing in wild-type and *ag1*-transgenic mice

**FIGURE 4:**
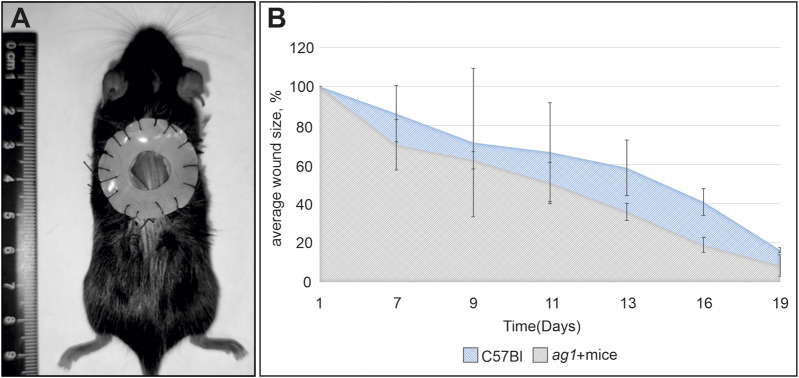
Analysis of Ag1 effects in *ag1* transgenic mice. **(A)** Schematic representation of the full-thickness fixed-splint skin wound model. **(B)** Effect of *ag1* expression on wound closure dynamics *in vivo* in transgenic versus control mice. The x-axis represents the days after the surgery (day 1) and the y-axis represents the percentage of the original wound size remaining. * marks the significant difference between experimental and control groups (p < 0.05).

A fixed full-thickness skin wound model was used to assess the effect of Ag1 on tissue regeneration. This approach enables a more accurate evaluation of re-epithelialization and tissue remodeling by minimizing the confounding effects of panniculus carnosus-mediated wound contraction in rodents (Figure 4A). In both control and ag^+^ mice, complete wound closure occurred within 18–19 days. Notably, on day 16, corresponding to the phase of active proliferation and final re-epithelialization, the residual wound area in ag^+^ mice was significantly smaller than in controls (18.93% vs. 40.88% of the initial wound size; *p* < 0.05) (Figure 4B). These results indicate that Ag1 expression accelerates the late stages of wound healing *in vivo*.

### Transcriptomic analysis reveals enhanced activation of wound-healing and regenerative genes in *ag1*-Expressing wound tissues

To assess the transcriptional effects of *ag1* expression during skin wound healing, we performed bulk RNA sequencing of wound tissues from three groups: (1) *ag1*-transgenic mice treated with doxycycline, (2) wild-type control mice, and (3) *ag1*-transgenic mice not treated with doxycycline. Each condition included three biological replicates. This experimental design enabled direct comparison of gene expression profiles between wound healing in transgenic mice with induced *ag1* expression and healing in the two control groups (Supplementary Table S1).

In these experiments RNA was isolated from wound tissue collected at the stage of complete re-epithelialization (recently closed wounds) in adult wild-type and ag1-transgenic mice. This time point was selected because it reflects the resolution phase of the wound healing process, when regenerative or fibrotic outcomes become most evident.

**FIGURE 5:**
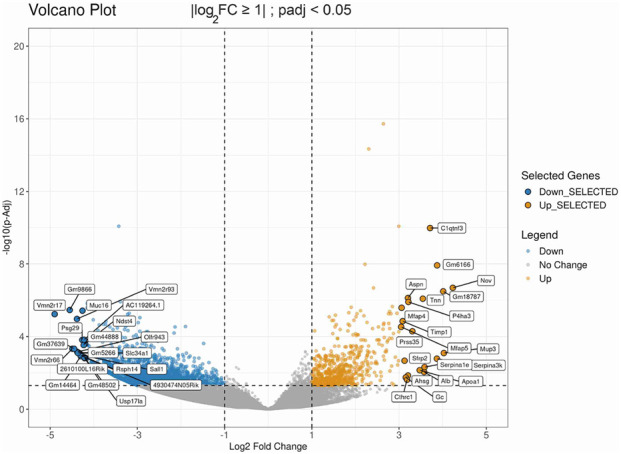
Transcriptomic impact of *ag1* expression in wounded skin. Volcano plot of differentially expressed genes in *ag1*-transgenic versus control mice. Genes with a fold change ≥2 or ≤ −2 (|log_2_ fold change| ≥ 1) and padj <0.05 were considered significantly regulated. Ginger and blue dots indicate downregulated activated genes, respectively. Grey dots indicate non-significant changes.

Genes that met the filtering criteria of |log_2_ fold change| ≥ 1 and padj <0.05 were considered significantly regulated. Compared with control samples, 2733 downregulated and 588 upregulated genes meeting these criteria were identified in transgenic mouse samples with induced *ag1* expression (Figure 5; Supplementary Table S1).

**FIGURE 6:**
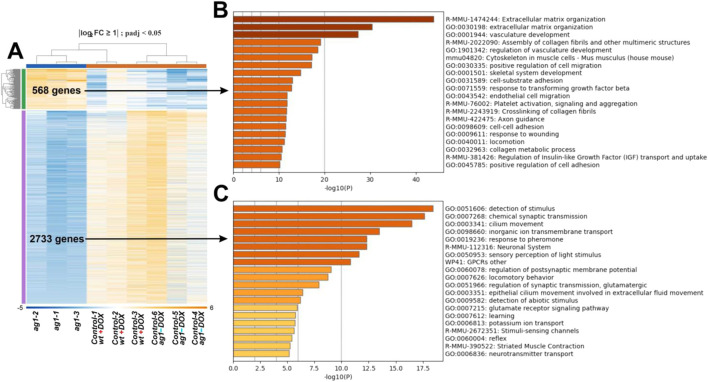
Differential gene expression and pathway enrichment in *ag1*-expressing wounds. **(A)** Heatmap of all differentially expressed genes (DEGs), showing clear clustering of biological replicates and distinct transcriptional profiles between *ag1*-transgenic and control wound tissues. **(B)** Enrichment analysis of downregulated DEGs identifies processes related to trans-synaptic signaling, excitatory signaling, such as trans-synaptic signaling, cilium movement, and response to calcium. **(C)** Enrichment analysis of upregulated DEGs highlights biological processes associated with tissue repair, including extracellular matrix organization, regulation of cell migration, vascular development, collagen biosynthesis, and wound response.

To further examine expression trends across samples, we generated a heatmap of all genes passing the filtering threshold (Figure 6A). This heatmap showed and distinct expression patterns between the *ag1*-expressing and control samples. The data indicated a reproducible transcriptomic response to *ag1* induction.

Pathway enrichment analysis of the genes differentially activated in mice with the induced *ag1* expression revealed a set of biological processes relevant to tissue repair (Figure 6B). Among the most significantly enriched pathways were extracellular matrix organization, positive regulation of cell migration, blood vessel development, vascular morphogenesis, supramolecular fiber organization, collagen biosynthesis, and wound response.

At the same time, among the genes differentially inhibited in the *ag1*-expressing mice were those associated with the following pathways, which in sum may indicate reduction in neuronal/sensory and excitatory signaling programs within wound tissue: nervous system development, detection of stimulus, response to calcium ione, protein-protein interactions at synapses, modulation of chemical synaptic transmission, cilium movement, detection of mechanical stimulus involved in sensory perception brain development, maintenance of synaptic structure, monoatomic ion transmembrane transport (Figure 6C).

**FIGURE 7:**
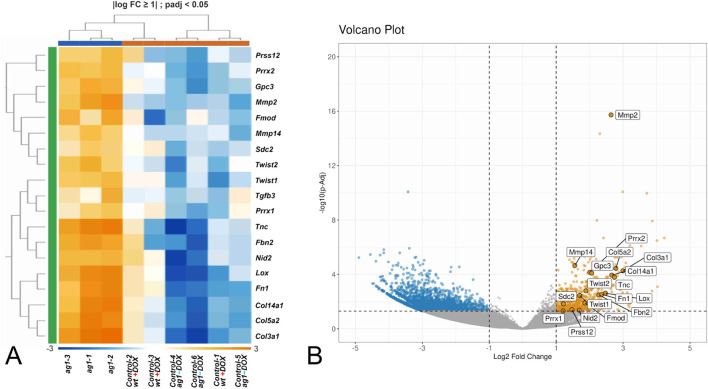
*Ag1* expression reactivates genes characteristic of scarless wound healing. **(A)** Heatmap showing overlap between upregulated genes in *ag1*-transgenic mice and a curated gene set associated with fetal scarless repair (see Supplementary Table S2). Hallmark regenerative genes such as *Col3a1* (*Collagen type III*), *Tgfβ3*, *Prrx1*, *Prrx2*, *Tnc* (*Tenascin-C*), *Twist1* and *Twist2* were consistently upregulated, mirroring fetal regeneration programs. **(B)** Positions of the genes shown in heatmap A on the volcano plot in Figure 5.

To explore the regenerative nature of the Ag1-induced transcriptome, we compared the list of upregulated genes in Ag1-transgenic mice to a curated set of genes known to be specifically activated during scarless wound healing in fetal skin (Supplementary Table S2). This comparison was visualized using a dedicated heatmap, which revealed substantial overlap between the two gene sets (Figure 7). Notably, genes such as Collagen type III, TGF-β3, Prrx1, Prrx2, Tenascin-C and others were consistently upregulated in Ag1-transgenic mice and are also hallmark markers of fetal regeneration (Leung et al., 2012; Lin et al., 1995; McKean et al., 2003; Seifert et al., 2012; Yokoyama et al., 2011).

Together, these results demonstrate that expression of the evolutionarily lost *ag1* gene in adult mouse skin reactivates transcriptional programs associated with scarless healing. The data suggest that *ag1* plays a regulatory role in re-establishing regenerative pathways that are otherwise inactive in postnatal mammalian wound repair.

## Discussion

In recent years, cross-species bioengineering has emerged as a powerful tool in both basic research and translational medicine, exemplified by technologies such as CRISPR-Cas systems (Cong et al., 2013; Jinek et al., 2012; Mali et al., 2013; Sharma et al., 2021), and by the successful expression of functional genes from non-mammalian species in mammalian cells and organisms (Fischer et al., 2001; Li et al., 2008; Looso et al., 2012; Szent-Györgyi et al., 2004; Qi et al., 2022).

In the present study, we observed partially accelerated skin wound healing in transgenic mice expressing the *ag1* gene from the frog *Xenopus laevis*, which was lost in the amniote lineage during evolution (Ivanova et al., 2013; Ivanova et al., 2015). Consistent with this *in vivo* effect, our *in vitro* scratch assays demonstrated enhanced migration of immortalized keratinocytes treated with recombinant Ag1, while no increase in proliferation rate was detected under these conditions. These observations suggest that the accelerated wound closure in *ag1*-expressing mice might be driven primarily by enhanced keratinocyte migration rather than proliferation. Nevertheless, additional experiments will be required to confirm this hypothesis conclusively.

Transcriptome profiling of regenerating tissues in transgenic mice revealed that the *ag1*-induced phenotype was accompanied by significantly increased expression of genes across multiple pathways involved in skin wound repair. The ten most strongly upregulated pathways included extracellular matrix organization, positive regulation of cell migration, blood vessel development, regulation of vascular development, supramolecular fiber organization, collagen formation, positive regulation of cell adhesion, response to wounding, and elastic fiber formation.

Notably, several of the upregulated genes are specifically associated with scarless healing, a feature of both fetal skin and amphibian regeneration. The most prominent among them include *Collagen type III*, *Tgfb3*, the matrix metalloproteinases *Mmp2* and *Mmp14*, the homeobox genes *Prrx1* and *Prrx2* (and their downstream target *Tenascin-C*), as well as *Twist2*.

In mammals, scarless fetal wound healing is characterized by a higher Collagen III to Collagen I ratio, which contributes to a more flexible, reticular extracellular matrix that facilitates cell migration and tissue remodeling (Leung et al., 2012). In contrast, adult scar formation is associated with reduced Collagen III expression and dense Collagen I deposition, resulting in fibrotic tissue (Cheng et al., 2014; Bullard et al., 2003). A similar shift in collagen composition has been observed in regeneration-competent amphibians such as the axolotl, where Collagen III is initially deposited and later replaced by Collagen I during the progression of healing (Seifert et al., 2012). This conserved elevation of the Collagen III to Collagen I ratio appears to be a fundamental feature of scar-free regeneration.

Remarkably, when we calculated the ratio of average normalized RNA-seq read counts for Collagen III to Collagen I in wound tissue samples, we observed a similarly pronounced shift favoring Collagen III expression in transgenic animals with activated *ag1* compared with control samples (Table 1). In addition, immunohistochemical staining of Collagen I and Collagen III using specific antibodies on histological sections of newly formed skin after wound healing (Supplementary Figures S1A, B) revealed a shift toward Collagen III in *ag1*-activated transgenic mice relative to controls (Supplementary Figure S1C).

**TABLE 1:** Ratio of normalized Collagen III to Collagen I and Tgfb3 to Tgfb1 RNA-seq read counts in control and ag1 transgenic wound tissue samples. Normalized RNA-seq read counts from 6 control and 3 *ag1* transgenic wound tissue samples (as listed in Supplementary Table S1) were used to calculate mean values and their ratios. As Collagen I is a heterotrimer composed of two α1 chains (encoded by *Col1a1*) and one α2 chain (encoded by *Col1a2*), data for both genes are shown separately.

Collagen	Mean value of normalized RNA-seq read counts in control wound samples	Mean value of normalized RNA-seq read counts in *ag1* transgenic wound samples
*Col1a1*	6,829	23,075
*Col1a2*	4,418	22,177
*Col3a1*	2,435	29,778
*Tgfb1*	128	169
*Tgfb2*	74	84
*Tgfb3*	74	181

*Col3/Col1* ratio	Mean collagen III/I read count ratio in control wound samples	Mean collagen III/I read count ratio in *ag1* transgens wound samples
*Col3a1/Col1a1*	0,3566	1,2905
*Col3a1/Col1a2*	0,5512	1,3427

*Tgfb3/Tgfb1* ratio	Mean Tgfb3/Tgfb1 read count ratio in control wound samples	Mean Tgfb3/Tgfb1 read count ratio in *ag1* transgens wound samples
*Tgfb3/Tgfb1*	0,58	1,07
*Tgfb3/Tgfb2*	1,00	2,15

In contrast to adult wound healing, which is dominated by profibrotic TGF-β1 and TGF-β2, fetal scarless repair is characterized by elevated TGF-β3 expression relative to TGF-β1 and TGF-β1 (Leung et al., 2012). This “reverse” ratio is considered a driving force in determining whether a tissue regenerates or forms a scar. Higher TGF-β3 levels reduce inflammation and drive organized matrix deposition, whereas high TGF-β1 and TGF- β1 promotes fibrosis (Lin et al., 1995; O’Kane and Ferguson, 1997). Administration of exogenous TGF-β3 in adult animals reduces scarring and improves collagen architecture, mimicking aspects of fetal repair (O’Kane and Ferguson, 1997; Gilbert et al., 2016). Although fewer studies exist in amphibians, available evidence suggests that regenerative species such as the axolotl may employ similarly non-fibrotic TGF-β dynamics (Gilbert et al., 2016).

Interestingly, similar to what we observed for Collagen III and Collagen I, the ratio of mean normalized RNA-seq read counts for *Tgfb3* and *Tgfb1* in Ag1-transgenic adult skin wound samples also showed a shift in favor of *Tgfb3* compared with control samples, a feature characteristic of scarless fetal skin healing (Table 1).

The homeobox genes *Prrx1* and *Prrx2* are likewise key regulators of scar-free healing. In amphibians, *prrx1* is reactivated during skin and limb regeneration and directly induces *Tenascin-C*, a matrix glycoprotein required for scar-free repair (McKean et al., 2003; Seifert et al., 2012; Yokoyama et al., 2011). In mammals, *Prrx2* is highly expressed in fetal fibroblasts and promotes matrix remodeling, proliferation, and hyaluronic acid synthesis—features largely absent in adult wound healing (Stelnicki et al., 1998; Dou et al., 2025).

*Twist2*, which was activated in *ag1*-expressing wounds compared with controls, is also essential for regenerative fetal skin repair, functioning in part by activating Wnt/β-catenin signaling and maintaining fibroblast plasticity (Takaya et al., 2024).

Together, these genes specifically associated with scarless healing appear to establish a molecular environment favorable to regeneration rather than fibrosis and thus represent promising candidates for therapeutic exploration.

Noteworthy, the enhanced expression of such genes as *Collagen III, TGF-β3, Twist2, Prrx1, Prrx2, and Tenascin-C* is known to be associated with fetal fibroblasts (Buechler et al., 2021; Srivastava et al., 2023; Morioka et al., 2025). Therefore, activation of them during adult mouse skin wound healing suggests that introduction of *ag1* in mouse genome may modulate the fibroblast transcriptional landscape, promoting a less differentiated, more progenitor-like state. Such a shift could facilitate regenerative rather than fibrotic responses, offering mechanistic insight into how fibroblast heterogeneity influences repair outcomes across developmental stages.

In addition to the activation of regenerative pathways, our transcriptomic analysis revealed that several neuronal and sensory-related pathways were suppressed in *ag1*-expressing wounds. These included synaptic signaling, calcium- and ion-dependent excitability, and sensory perception programs. Such downregulation may indicate a dampening of neuronal-like gene expression in wound tissues, which is of particular interest given the emerging role of the nervous system in regulating inflammation and fibrosis during skin repair. Neurogenic inflammation mediated by sensory neurons and calcium-dependent excitatory signaling has been implicated in excessive fibroblast activation and scar formation (Roosterman et al., 2006; Ji et al., 2016). Conversely, inhibition of these pathways may reduce myofibroblast differentiation and attenuate fibrotic remodeling (Mauck et al., 2014; Xu et al., 2022). Thus, suppression of excitatory and neurogenic signaling in *ag1*-expressing mice could contribute to creating a more permissive, scarless-like wound environment, complementing the pro-regenerative gene expression signature described above.

Intriguingly, however, we also detected increased expression of a suite of genes typically associated with scar-forming adult wound repair. These included *Acta2* (α-smooth muscle actin), *Tinagl1*, *Thbs3*, *Tgfbr2* and numerous chemokines involved in myofibroblast differentiation and fibrosis, such as *Cxcl12*, *Ccl7*, *Ccl8*, and *Ccl21a* (Bergmeier et al., 2018; Cowin et al., 2001; Vorstandlechner et al., 2021). Moreover, we observed a striking increase in four isoforms of type VI collagen (*Col6a1*, *Col6a2*, *Col6a3*, *Col6a4*), which are normally enriched at the borders of keloid scars (Theocharidis et al., 2016).

This combination of activation of genes associated with both regeneration and fibrosis suggests that *ag1* expression does not result in completely scarless healing in adult mice, as observed in fetal skin. Instead, it appears to trigger a complex hybrid program involving both regeneration-promoting and scar-promoting elements. Supporting this interpretation, we found no significant morphological differences in histological sections of wound tissue from *ag1*-positive and control mice, either by standard hematoxylin and eosin staining (Supplementary Figure S2A) or by van Gieson staining, which allows visualization of collagen fibers, including the red-stained fibers in the periwound dermis (Supplementary Figure S2B).

At present, we do not yet know the specific mechanisms by which transgenic Ag1 exerts the effects described above. However, as a first approximation, we may consider existing data on another member of the Agr family, Agr2, which shares 57% amino acid identity with Ag1 and is co-expressed with it during amphibian and fisch body appendages regeneration (Novoselov et al., 2003; Ivanova et al., 2013; Ivanova et al., 2015). Because Agr2 is also a known marker of various human cancers, it has been studied in greater detail than Ag1, which is specific to anamniotes (Obacz et al., 2015). Like Ag1, Agr2 is a multidomain protein consisting of five domains. This structural organization enables interactions with multiple signaling pathways and allows it to function both as a secreted signaling molecule and as an ER-resident chaperone via its C-terminal ER-retention motif (Delom et al., 2020; Ullah et al., 2025). During newt limb regeneration, Agr2 interacts with the three-finger protein Prod1 and EGFR, regulating the formation of the wound epidermis and the blastema (Blassberg et al., 2011). In this context, Agr2 promotes cell proliferation. In mammalian systems, Agr2 also modulates the activity of various receptors and ligands, including CD59, EpCAM, FGF, and VEGF (Kosykh et al., 2023).

Interestingly, in contrast to the ability of Ag1 to increase Collagen III expression, as demonstrated in the present study, Agr2 (nAG) from the newt *Notophthalmus* sp. has been shown to suppress the synthesis of both Collagen I and III and to increase collagen degradation in cultured human fibroblasts (Al-Qattan et al., 2013).

Given the diversity of interactions documented for Agr2 it is plausible that the complex effects of its homolog Ag1 also result from its ability to engage multiple signaling pathways. Moreover, it may not be the presence of these interactions alone, but rather the specific combination and intensity of their activation, that is critical for triggering a regenerative skin response similar to those seen in fetal mammals and amphibians. This unique configuration of Ag1-mediated signaling might act as a molecular code for initiating scarless healing programs, which cannot be replicated by targeting individual pathways in isolation. This may explain why attempts to enhance regeneration by manipulating individual growth factors or signaling cascades have yielded limited success (Nacu and Tanaka, 2011; Mathew et al., 2022). As such, Ag1 may offer a more effective means of activating regenerative responses than any single pathway-specific factor or combination thereof. Experimental validation of this hypothesis represents an important future direction. First of all, future experiments should identify with which mammalian pathway components Ag1 can physically interact to orchestrate a regenerative transcriptomic profile.

## Materials and methods

### Animals

#### Ethics statement and animal husbandry

All animal procedures were approved by the Committee for the Control of the Care and Use of Laboratory Animals of the Pirogov Russian National Research Medical University. Mice were housed with *ad libitum* access to food and water, under a 12 h light/12 h dark cycle, at 23 °C ± 1 °C and 42% ± 5% humidity.

#### Commercial transgenic mouse strains

All transgenic mouse lines were obtained from The Jackson Laboratory unless stated otherwise. B6.129-Gt (ROSA)26Sortm1 (cre/ERT2) Tyj/J (Stock No. 008463; R26-CreERT2) and B6.Cg-Gt (ROSA)26Sortm2(CAG-rtTA3-mKate2) Slowe/J (Stock No. 029633; rtTA transactivator) were used for transgene regulation. C57BL/6 mice from the Pushchino Animal Facility (Russia) served as controls. To standardize genetic backgrounds across sources, transgenic lines were backcrossed to C57BL/6 for multiple generations.

#### Generation of F0 and F1 *ag1* ^+^ transgenic mice

Transgenic mice carrying the *ag1* cassette were produced by pronuclear microinjection of the pKB2-ag1 plasmid into fertilized C57BL/6 eggs. In total, 1,227 embryos were injected and implanted into 93 pseudopregnant females, resulting in 96 pups derived from 17 recipients. PCR genotyping identified three founder (F0) mice positive for the *ag1* transgene (Supplementary Figure S3).

Founder males were crossed with wild-type C57BL/6 females, producing seven F1 *ag1*-positive offspring, confirming germline transmission of the transgene. Five F1 *ag1*-positive males were retained for subsequent breeding. In parallel, B6.129-Gt (ROSA)26Sortm1 (cre/ERT2)Tyj/J (R26-CreERT2, Stock No. 008463) mice were crossed with B6.Cg-Gt (ROSA)26Sortm2(CAG-rtTA3,-mKate2)Slowe/J (rtTA, Stock No. 029633) mice, generating a double-transgenic Cre/rtTA line that carries both regulatory elements required to activate *ag1* expression. To generate Cre/rtTA/*ag1* triple-transgenic animals for conditional induction experiments, the target transgenic line, F1 *ag1*-positive mice were then crossed with Cre/rtTA mice, and the resulting offspring were screened by PCR to identify individuals carrying all three transgenes (*Cre*, *rtTA*, and *ag1*) (Supplementary Figure S4). Excision of the Stop cassette was verified by PCR on tail biopsy DNA using primers Fw-TermC, RG-TermC, FW-STOP, RV-STOP (Supplementary Figure S5) (primers are shown in Supplementary Table S3). After confirming that Cre-mediated removal of the stop cassette caused no adverse effects in the tamoxifen-induced founder animals, the male mice were crossed with C57 females. The F1 offspring with rtTA, Δstop ag1/^WT^ genotype was subsequently backcrossed to generate mice with the desired genotype (Δstop *ag1, rtTA)*, selecting for homozygosity where verification was possible. These mice were then used in the series of skin wound healing model experiments described below.

#### Conditional *ag1* expression in mice

Expression was induced with doxycycline (Sigma-Aldrich, D9891) in drinking water (200 μg/mL) for 7 days. RNA from tail biopsies was isolated using the RNeasy Mini Kit (Qiagen), reverse-transcribed (High-Capacity cDNA Reverse Transcription Kit, Applied Biosystems), and analyzed by RT-PCR. Mice with confirmed *ag1* expression on day 7 were used for subsequent experiments.

### Plasmid vector and lentiviral construction

#### Plasmid vector for generation of *ag1*-expressing transgenic mice

*Ag1* cDNA was amplified using FW-ag1Age1 and RV-ag1Mlu1 STOP primers from *ag1*-*pCS2* plasmid described in (Ivanova et al., 2021). The resulting plasmid *pKB2-ag1* (Supplementary Figure S6) contains a Tet-On promoter with seven tet operator sequences, activated by tetracycline/doxycycline. A Stop-cassette containing an intron and nuclear localization signal separates the *ag1* fragment from the Tet-On promoter. In the presence of the Stop-cassette, ag1 expression does not occur. Two LoxP sites flank the Stop-cassette, enabling Cre recombinase–mediated excision. The plasmid includes SV40polyA and PMSC SMARterm as polyadenylation and transcription termination signals. The ampicillin resistance gene (AmpR) is shown in green, the plasmid origin of replication (ori) in yellow, the HS4 insulator in blue, and the SKIV 2L_DXO sequence in white as an additional insulator.

#### *Ag1* lentiviral plasmid construction and virus production

Lentiviral particles were produced in HEK293T cells using a second-generation packaging system. The transfer vector pLVH-EF1a-ag1-T2A-TurboFP was constructed from the parental plasmid pLVH-EF1a-BRD3R (Addgene plasmid #130696; RRID: Addgene_130696) and pTurboFP635-C vector (Evrogen, Moscow, cat # FP721) using standard molecular cloning techniques. One day prior to transfection, 1.5 × 10^6^ HEK293T cells were seeded in 60-mm dishes containing 3 mL of DMEM supplemented with 10% fetal bovine serum (FBS). Immediately before transfection, the medium was replaced with 1.3 mL Opti-MEM. A transfection mixture was prepared by incubating 350 μL Opti-MEM with 20 μL FuGENE-6 reagent (Promega) for 5 min (mixture 1). In parallel, 2 μg of pLVH-EF1a-ag1-T2A-TurboFP, 2 μg of pR8.91, and 0.6 μg of pMD.G were diluted in 350 μL Opti-MEM and then gently added to mixture 1. After 10 min incubation, the final transfection mix was added dropwise to the cells. Virus-containing supernatants were collected 48 h post-transfection, passed through a 0.45-μm filter, and used immediately to transduce target cells.

#### *Ag1* expression in human cell cultures

Immortalized human keratinocytes (immNHK) and fibroblasts (immNHF) were transduced with lentiviral particles carrying pLVH-EF1a-ag1-T2A-TurboFP. Following expansion, successfully transduced cells were confirmed by TurboFP fluorescence microscopy.

### Recombinant Ag1 protein and production of Ag1 polyclonal antibodies

His-tagged Ag1 protein was produced and purified as described previously (Ivanova et al., 2021). The purified protein was used both in *in vitro* experiments on immortalized keratinocytes and as an antigen for rabbit immunization. Polyclonal antiserum was affinity-purified using antigen-conjugated resin according to Martynova et al. (2008). For resin preparation, recombinant Ag1 protein containing an N-terminal His-tag was covalently coupled to cyanogen-bromide–activated Sepharose (Sigma, C9210) following the manufacturer’s protocol. The serum was applied to the Ag1-Sepharose column, washed with 100 mM boric acid, 75 mM NaCl (pH 8.4), and specific antibodies were eluted with 0.1 M glycine-HCl (pH 2.7) and immediately neutralized with 1 M Tris-HCl (pH 9.0). Antibody specificity was confirmed by immunoblotting.

### Full-thickness skin wound healing model

All experiments were performed using 12-week-old male mice weighing 25–28 g. One week before surgery, all mice, including C57BL/6J controls, received doxycycline (200 μg/mL in drinking water) to induce ag1 expression and control for possible antibiotic effects. Animals were weighed and anesthetized with intraperitoneal 2,2,2-tribromoethanol (250 mg/kg) and given carprofen (5 mg/kg) for analgesia. An 8 mm circular wound was created on the dorsal surface between the scapulae using a biopsy punch and surgical scissors. A silicone splint with an opening matching the wound diameter was positioned, secured with sutures at 3 mm intervals, and covered with Tegaderm for 24 h. Each experimental group contained five animals, and the procedure was repeated in three independent experiments. After complete wound closure, newly formed skin of the same size was excised post-euthanasia for analysis.

### Cell culture and *in vitro* assays

#### Cell cultures

All cell cultures were maintained at 37 °C in a humidified atmosphere containing 5% CO_2_ (Sanjo incubator MCO-20AIK, Japan) in DMEM supplemented with 2 mM glutamine and 10% serum.

#### Cell proliferation assay

Proliferation of HaCaT cells and primary human fibroblasts was assessed after 48 h of incubation with Ag1 protein. Control cells received an equivalent volume of elution/dissolving buffer. Cells were fixed in 4% paraformaldehyde in PBS (pH 7.4) for 15 min at room temperature and stained with anti-Ki67 antibodies (Rb monoclonal, ab16667) and Donkey anti-Rabbit IgG, Alexa Fluor Plus 488 (A32790TR). The proliferation index was calculated as the number of Ki67-positive cells divided by the total number of DAPI-positive nuclei. The experiment was independently repeated twice, with each group plated in three wells (N = 6). Imaging was conducted using an EVOS FL AUTO (Life Technologies) fluorescence microscope. For each sample, three random images were captured, and nuclei were counted semi-automatically in ImageJ software.

#### *In vitro* scratch assay

After removing a strip of the cell monolayer, Ag1 protein (5 ng/μL) was added to the medium of the experimental group, while control cultures received elution/dissolving buffer. Wound closure was recorded over 48 h at 37 °C and 5% CO_2_ using EVOS FL AUTO (Life Technologies) microscope with time-lapse imaging. The experiment was independently repeated twice, control group plated in 3 wells, HaCaT + Ag1 group plated in 6 wells.

The linear mixed-effects model with the formula healing ∼ group * time + (1 | sample) was used to analyze wound-healing trajectories between control (HaCaT) and experimental (HaCaT + Ag1) groups. It included fixed effects for group (control vs. experimental), time, and their interaction group:time, and a random intercept for sample to account for repeated measures. ANOVA-like type III F tests with Satterthwaite’s degrees of freedom evaluated each term’s contribution and showed a significant main effect of group (F (1, 29.04) = 67.67, p = 4.49 × 10^-9), time (F (1, 421) = 746.60, p < 2.2 × 10^-16) and the group-by-time interaction (F (1, 421) = 40.27, p = 5.73 × 10^-10). The data with the wound-healing measurements across samples, linear mixed-effects model and type III F tests results are provided in Supplementary Table S4.

### Molecular methods

#### RNA extraction and quantitative real-time polymerase chain reaction (RT-qPCR)

Tissue samples were lysed in Qiazol (Quiagen, Germany), flash-frozen in liquid nitrogen, and mechanically ground. RNA was extracted via two sequential chloroform incubations followed by isopropanol precipitation. We then assessed RNA quality and quantity with a NanoPhotometer P360 (Implen, Germany) and agarose gel electrophoresis. Finally, 1 μg of total RNA was used as the template for cDNA synthesis.

To eliminate genomic DNA, samples were treated with the RNase-free DNase Set (Qiagen, Germany) according to the manufacturer’s instructions. First-strand cDNA was synthesized using MMLV reverse transcriptase (Eurogen) and a random primer. Quantitative RT-PCR (RT-qPCR) was performed using a CFX96™ Real-Time System (Bio-Rad). Reaction mixtures were prepared with HS-SYBR master mix (Evrogen, Russia). The temperature profile comprised three steps: an initial 10-min incubation at 95 °C, followed by 40 cycles of denaturation at 95 °C for 15 s, and annealing/extension at 60 °C for 1 min. Subsequently, a melt curve analysis was performed within the temperature range of 60 °C–95 °C. Three reference genes, GAPDH, ODC, and EF1, were selected for normalization of target gene expression. Specific primer sequences used for RT-qPCR are listed in Supplementary Table S3. Each gene was analyzed with three independent biological replicates, each with technical triplicates. Gene expression was calculated using the 2^−ΔΔCT^ method. The cycle threshold (Ct) values from the three independent experiments were averaged to determine the mean Ct and standard deviation. Statistical significance of the observed differences was assessed using analysis of variance (ANOVA). Sequences of primers for qPCR was shown in Supplementary Table S3.

#### High-throughput sequencing and bioinformatic analysis

RNA quality was verified with a Bioanalyzer and RNA 6000 Nano Kit (Agilent). Poly(A) RNA was purified with the Dynabeads® mRNA Purification Kit (Ambion). Libraries were prepared from poly(A) RNA using the NEBNext® Ultra™ II RNA Library Prep Kit for Illumina (NEB). Sequencing was performed on an Illumina HiSeq1500 platform with 50 bp single-end reads, generating at least 30 million reads per sample. Accession number of raw RNA-seq data in the GEO database is GSE315128.

FASTQ files were processed using the ROSALIND platform (https://rosalind.bio/, San Diego, CA). Adapter trimming was carried out with Cutadapt, and reads were aligned to the *Mus musculus* reference genome build GRCm39 using STAR. The percentage of aligned reads after trimming was ≥88%. Read counts were obtained with HTSeq4 and normalized by Relative Log Expression (RLE) with the DESeq2 R package. DESeq2 was also used to calculate fold changes, p-values, and perform optional covariate correction. Differentially expressed genes (DEGs) were defined as those with |log_2_ fold change| ≥ 1 and padj <0.05. Clustering of DEGs for heatmap visualization was performed using the Partitioning Around Medoids (PAM) algorithm implemented in the *fpc* R package.

Pathway enrichment analysis was conducted with Metascape (https://metascape.org) using default parameters (minimum overlap = 3, p-value cut-off = 0.01, minimum enrichment = 1.5).

#### Tissue collection, fixation, and histological processing

Skin biopsies were collected after complete wound closure, on day 20 post-wounding. Biopsy specimens consisted of a skin fragment encompassing the former wound area together with adjacent intact tissue. Immediately after excision, samples were immersed in an excess volume of 10% neutral buffered formalin (NBF), maintaining a tissue-to-fixative ratio of at least 1:10, and fixed for 24–48 h at room temperature. Following fixation, tissue samples were the skin surface and passed through the center of the former wound area as well as the adjacent intact skin. Samples were then processed for paraffin embedding using standard histological procedures. Briefly, tissues were dehydrated through a graded ethanol series (70%, 80%, 90%, and 96% ethanol for 1–2 h each), followed by two changes of 100% ethanol for 1–1.5 h each to achieve complete dehydration.

#### Immunohistochemical staining and quantification of collagen I and collagen III

For immunohistochemical analysis, skin sections were deparaffinized, subjected to antigen retrieval in citrate buffer (pH 6.0), and blocked with serum and hydrogen peroxide. Sections were then incubated overnight with mouse primary antibodies against Collagen I (1:500 dilution, GB114197, Servicebio) or Collagen III (1:250 dilution, GB111629, Servicebio). Primary antibodies were detected using horseradish peroxidase–conjugated anti-rabbit secondary antibodies (1:1000, S0001, Affinity) and the DAB (3,3′-diaminobenzidine) substrate. Sections were counterstained with hematoxylin to visualize nuclei.

Immunohistochemical staining was performed in duplicate for each experimental group. Staining for Collagen I and Collagen III was carried out simultaneously to minimize technical variability. For each section, three randomly selected fields of view were analyzed (18 fields in total for each experimental group and each type of staining). Matrix staining intensity was quantified using ImageJ software (NIH) following color deconvolution and normalized to the number of nuclei per field.

All images were acquired using a Nikon Eclipse Ni upright microscope equipped with a DS-Ri2 camera (Nikon Corporation, Japan).

## Data Availability

The datasets presented in this study can be found in online repositories. The names of the repository/repositories and accession number(s) can be found in the article/Supplementary Material.
