# Supplementary material for: Restoring Ag1, an ancient regeneration gene lost in amniotes, accelerates skin healing in mice
Source: Front Cell Dev Biol. 2026 Feb 19;14:1706902. doi: 10.3389/fcell.2026.1706902 (PMC12960510; doi:10.3389/fcell.2026.1706902)
Supplement: Supplementary file 1 [file Table3.doc]

**Supplementary Table S2 List of primers used in the work**

| FW-xAG1Age1 | CAGCTACCGGTATGCAGACTGGCCTGTCACTTGCTTGCCTTGTC |
| --- | --- |
| RV-xAG1Mlu1 STOP | GTTCACGCGTCTAAAGCTCAGTCTTCAGGAAACTTTTAGC |
| FW-Cre | GCGGTCTGGCAGTAAAAACTATC |
| RV-Cre | GTGAAACAGCATTGCTGTCA CTT |
| FW-Rtta | ACACCGGCCTTATTCCAAG |
| RV-Rtta | CTTGATATGCTGCCTGCTGA |
| FW-xAG-RT | ATGTTGAATTTGGTGCATCC |
| RV-xAG-RT | GTAATCAACTCAGGAATATC |
| FW-GAPDH | TGGTGAAGCAGGCATCTGAG |
| RV-GAPDH | TCGAAGGTGGAAGAGTGGGA |
| FW-ODC | AGACCCAAGCCAGACGAGAA |
| RV-ODC | ACAGCATCCAATCACCCACA T |
| FW-EF1 | ATGGCCCCAAGTTCCTGAAG |
| RV-EF1 | ATGTCACGAACAGCAAAGCG |
| FW-TermC | GCGAGTCCATGTCACTCAGG |
| RV-TermC | GTGTTGCCCTTTGGAGCTTG |
| FW-STOP | GTTAGATCTGCTGCCACCGT |
| RV-STOP | AGGTGGCAAGTGGTATTCCG |
