# Supplementary material for: Restoring Ag1, an ancient regeneration gene lost in amniotes, accelerates skin healing in mice
Source: Front Cell Dev Biol. 2026 Feb 19;14:1706902. doi: 10.3389/fcell.2026.1706902 (PMC12960510; doi:10.3389/fcell.2026.1706902)
Supplement: Supplementary file 2 [file DataSheet1.PDF]

Supplementary Figures S1-S6

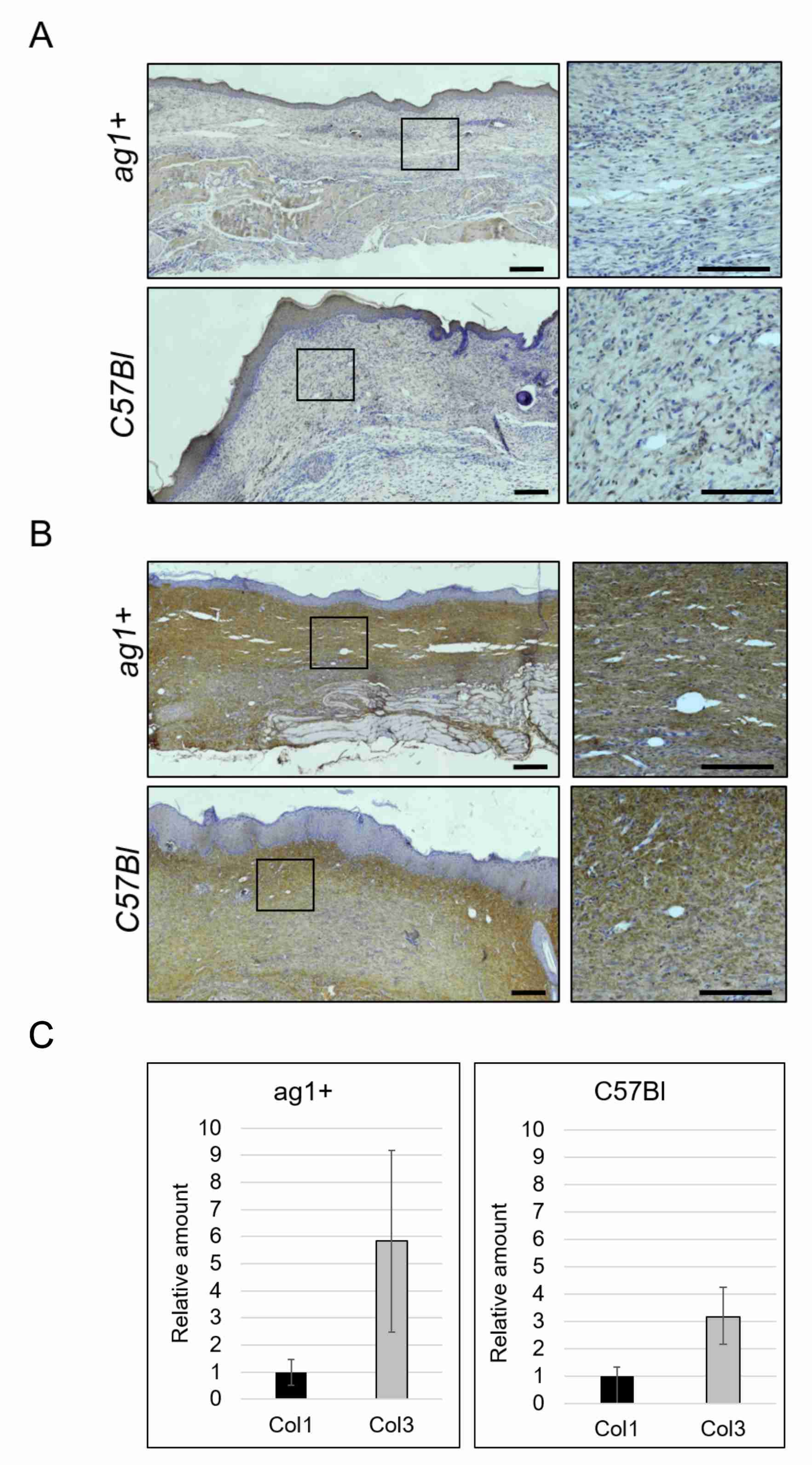

Figure S1. Immunohistochemical analysis of Collagen I and Collagen III deposition in post-wound skin of ag1-positive and control mice.
(A, B) Immunohistochemical staining for Collagen I (A) and Collagen III (B) in skin sections collected after wound closure. Representative micrographs from ag1-positive and control mice are shown at low (left column) and higher (right column) magnification. Scale bar = 200 μm. (C)

Semi-quantitative analysis of the Collagen III to Collagen I ratio (COL3:COL1) in post-wound skin sections from *ag1*-positive (left) and control (right) animals, based on immunohistochemical staining with type-specific antibodies (see Materials and Methods for details).

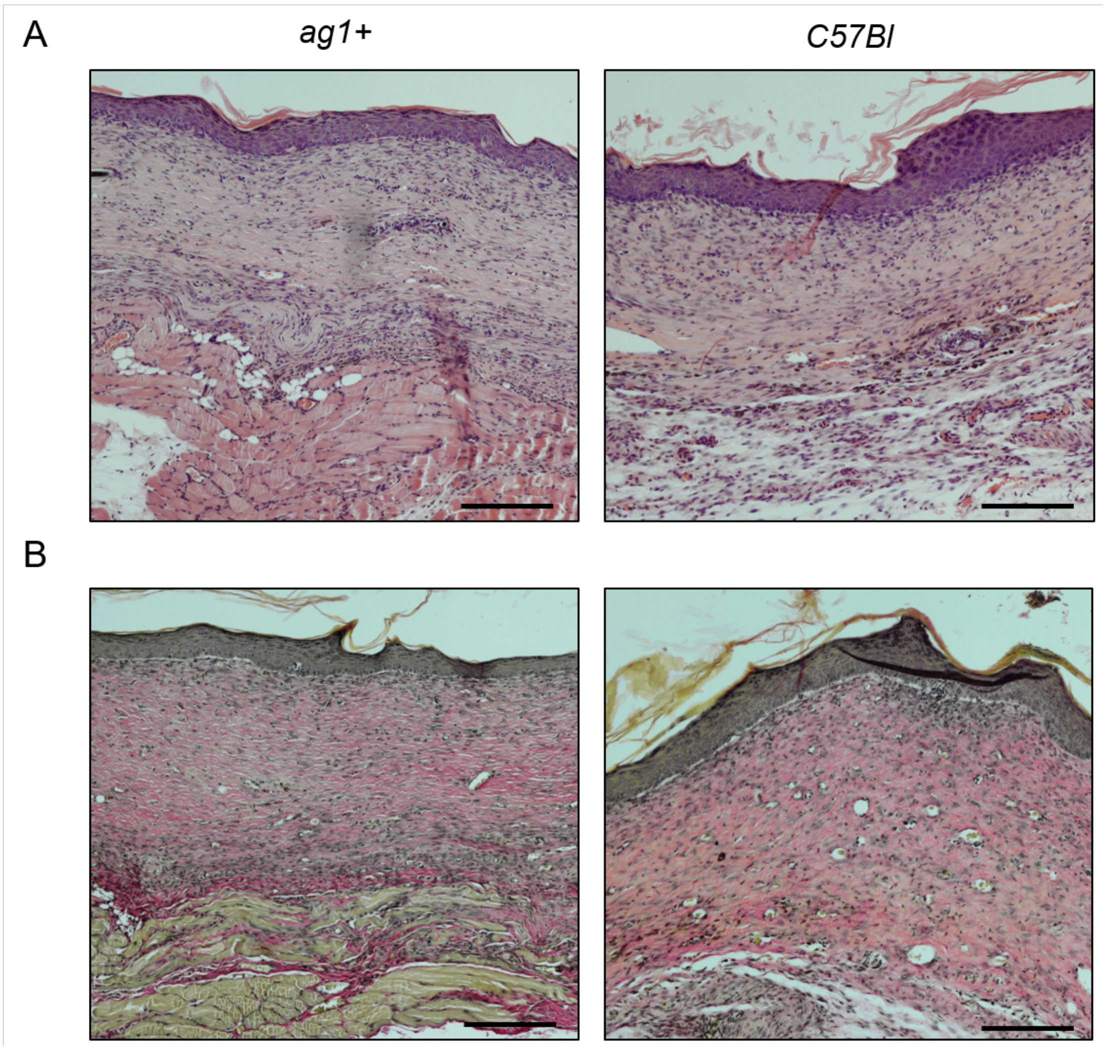

Figure S2. Histological assessment of wound tissue architecture in ag1-positive and control mice.
(A) Hematoxylin and eosin and (B) van Gieson staining of skin sections collected after wound closure. Representative micrographs from ag1-positive and control mice are shown. Scale bar = 100 µm.

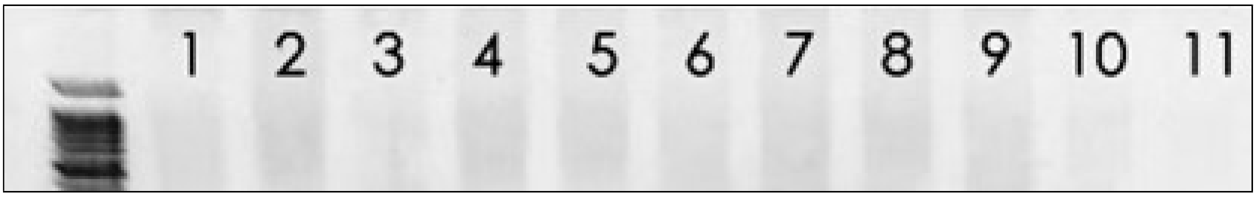

Figure S3. PCR genotyping of F0 founder mice using primers specific for *ag1* cDNA (270 bp amplicon).

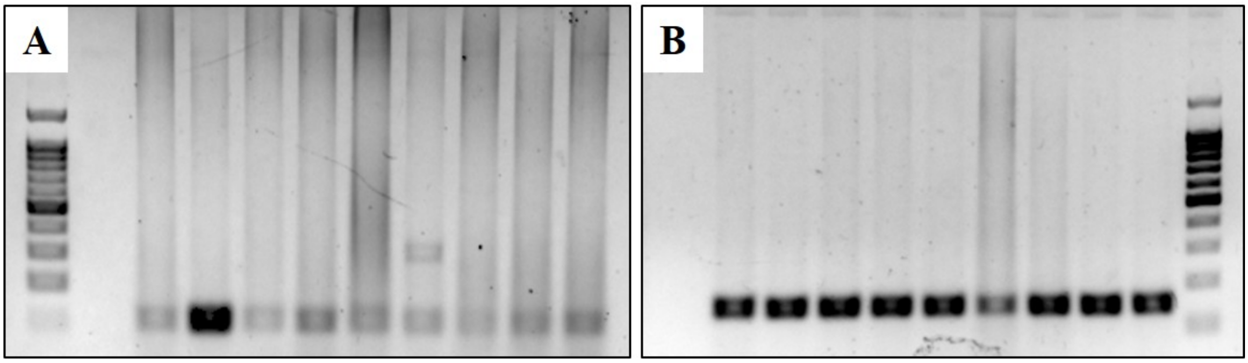

Figure S4. PCR screening of transgenic mouse tissues. (A) Detection of the *Cre* gene using specific primers (expected amplicon size: 100 bp). (B) Detection of *rtTA* using specific primers (expected amplicon size: 150 bp).

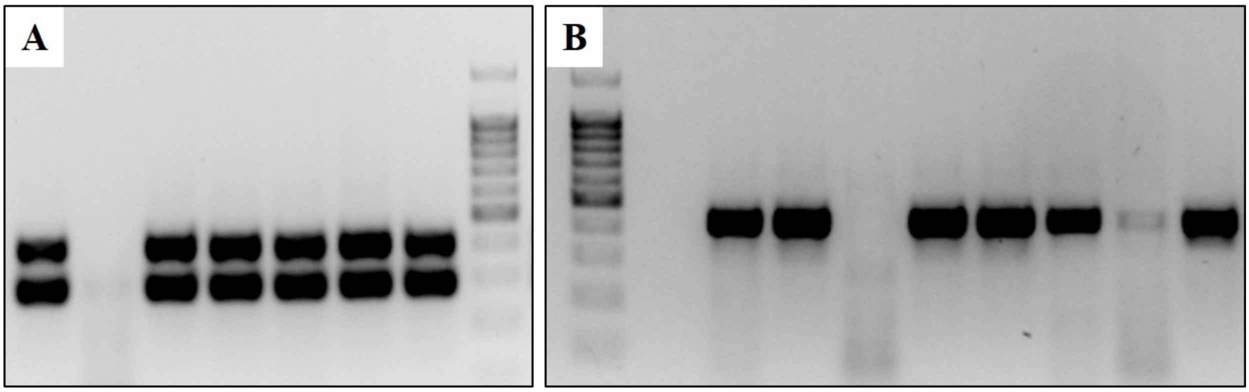

Figure S5. PCR evaluation of Stop-cassette excision in transgenic mice. Multiplex PCR was performed using primers specific for the Stop cassette (300 bp) and for the Terminator region (420 bp) in mice carrying *Cre*, *rtTA*, and *ag1*. (A) Two bands (300 bp and 420 bp) in untreated animals indicate preservation of the transgene construct and absence of recombination. (B) Loss of the 300 bp Stop-cassette band following tamoxifen treatment confirms successful Cre-mediated excision.

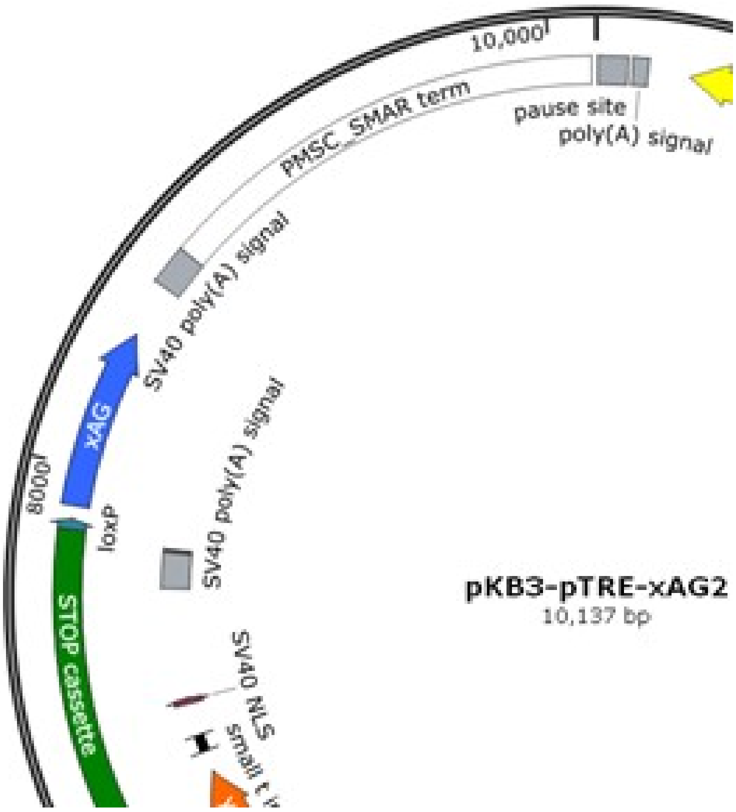

Figure S6. Map of pKB2Ag1 plasmid encoding ag1 under Tet-On promoter.
