# Supplementary material for: Restoring Ag1, an ancient regeneration gene lost in amniotes, accelerates skin healing in mice
Source: Front Cell Dev Biol. 2026 Feb 19;14:1706902. doi: 10.3389/fcell.2026.1706902 (PMC12960510; doi:10.3389/fcell.2026.1706902)
Supplement: Supplementary file 5 [file Table2.doc]

**Table S2. Genes specifically activated during fetal skin wound healing**

| Short name | Full name | Mouse gene NCBI ID | Species,  in which gene fetal specificity  was tested | Reference |
| --- | --- | --- | --- | --- |
| Col3a1 | Collagen, type III, alpha 1 | 12825 | Mouse | Castillo et al., 2023  Volk et al., 2011 |
| Col5a2 | Collagen, type V, alpha 2 | 12832 | Human | Chang et al., 2002 |
| Col14a1 | Collagen, type XIV, alpha 1 | 12818 | Human | Ramelet et al., 2009 |
| Fbn2 | Fibrillin 2 | 14119 | Human | Ramelet et al., 2009 |
| Fgf2 | Fibroblast growth factor 2 | 14173 | Rat | Chen et al., 2007 |
| Fgf7 | Fibroblast growth factor 7 | 14178 | Mouse | Takaya et al., 2022 |
| Fgf8 | Fibroblast growth factor 8 | 14179 | Rat | Chen et al., 2007 |
| Flt (Vegfr1) | FMS-like tyrosine kinase 1 | 14254 | Human | Colwell et al., 2005 |
| Fmod | Fibromodulin | 14264 | Rat | Soo et al., 2000 |
| Fn | Fibronectin | 14268 | Human | Coolen et al., 2010 |
| Gpc3 | Glypican 3 | 14734 | Human | Ramelet et al., 2009 |
| HIF1a | Hypoxia inducible factor 1, alpha subunit | 15251 | Sheep | Scheid et al., 2002 |
| Il10 | Interleukin 10 | 16153 | Mouse | Liechty et al., 2000 |
| Kdr (Vegfr2) | Kinase insert domain protein receptor | 16542 | Human | Colwell et al., 2005 |
| Lox | Lysyl oxidase | 16948 | Mouse | Colwell et al., 2006 |
| Mmp1a | Matrix metallopeptidase 1a | 83995 | Rat | Dang et al., 2003 |
| Mmp2 | Matrix metallopeptidase 2 | 17390 | Rat | Dang et al., 2003 |
| Mmp3 | Matrix metallopeptidase 3 | 17392 | Rat | Peled et al., 2002 |
| Mmp9 | Matrix metallopeptidase 9 | 17395 | Mouse | Colwell et al., 2008 |
| Mmp13 | Matrix metallopeptidase 13 | 17386 | Human/Mouse | Ravanti et al., 2001 |
| Mmp14 | Matrix metallopeptidase 14 | 17387 | Mouse | Dang et al., 2003 |
| Nid2 | Nidogen 2 | 18074 | Human | Ramelet et al., 2009 |
| Prss12 | Serine protease 12 neurotrypsin | 19142 | Human | Ramelet et al., 2009 |
| Prrx1 | Paired related homeobox 1 | 18933 | Human | Dou et al., 2025 |
| Prrx2 | Paired related homeobox 2 | 98218 | Mouse | White et al., 2003 |
| Sdc2 | Syndecan 2 | 15529 | Human | Chang et al., 2002 |
| TGFb3 | Transforming growth factor, beta 3 | 21809 | Human | Walraven et al., 2015 |
| Tnc | Tenascin-C | 21923 | Sheep | Whitby et al., 1991 |
| Twist1 | Twist basic helix-loop-helix transcription factor 1 | 22160 | Mouse,  Speeny mouse | Harn et al., 2021 |
| Twist2 | Twist basic helix-loop-helix transcription factor 2 | 13345 | Mouse | Takaya et al., 2024 |
| Vegfa | Vascular endothelial growth factor A | 22339 | Rat | Colwell et al., 2005 |

**References**

Castillo V, Díaz-Astudillo P, Corrales-Orovio R, San Martín S, Egaña JT. Comprehensive Characterization of Tissues Derived from Animals at Different Regenerative Stages: A Comparative Analysis between Fetal and Adult Mouse Skin. Cells. 2023 Apr 22;12(9):1215. doi: 10.3390/cells12091215.

Chang HY, Chi JT, Dudoit S, Bondre C, van de Rijn M, Botstein D, Brown PO. Diversity, topographic differentiation, and positional memory in human fibroblasts. Proc Natl Acad Sci U S A. 2002 Oct 1;99(20):12877-82. doi: 10.1073/pnas.162488599.

Chen W, Fu X, Ge S, Sun T, Sheng Z. Differential expression of matrix metalloproteinases and tissue-derived inhibitors of metalloproteinase in fetal and adult skins. Int J Biochem Cell Biol. 2007;39(5):997-1005. doi: 10.1016/j.biocel.2007.01.023.

Colwell AS, Beanes SR, Soo C, Dang C, Ting K, Longaker MT, Atkinson JB, Lorenz HP. Increased angiogenesis and expression of vascular endothelial growth factor during scarless repair. Plast Reconstr Surg. 2005 Jan;115(1):204-12. PMID: 15622252.

Colwell AS, Krummel TM, Longaker MT, Lorenz HP. Fetal and adult fibroblasts have similar TGF-beta-mediated, Smad-dependent signaling pathways. Plast Reconstr Surg. 2006 Jun;117(7):2277-83. doi: 10.1097/01.prs.0000224299.16523.76.

Colwell AS, Longaker MT, Peter Lorenz H. Identification of differentially regulated genes in fetal wounds during regenerative repair. Wound Repair Regen. 2008 May-Jun;16(3):450-9. doi: 10.1111/j.1524-475X.2008.00383.x.

Coolen NA, Schouten KC, Middelkoop E, Ulrich MM. Comparison between human fetal and adult skin. Arch Dermatol Res. 2010 Jan;302(1):47-55. doi: 10.1007/s00403-009-0989-8.

Dang, Catherine M. M.D.; Beanes, Steven R. M.D.; Lee, Haofu D.D.S.; Zhang, Xinli M.D., Ph.D.; Soo, Chia M.D.; Ting, Kang D.M.D., D.M.Sc.. Scarless Fetal Wounds Are Associated with an Increased Matrix Metalloproteinase–to–Tissue-Derived Inhibitor of Metalloproteinase Ratio. Plastic and Reconstructive Surgery 111(7):p 2273-2285, June 2003. | DOI: 10.1097/01.PRS.0000060102.57809.DA

Harn HI, Wang SP, Lai YC, Van Handel B, Liang YC, Tsai S, Schiessl IM, Sarkar A, Xi H, Hughes M, et al. 2021. Symmetry breaking of tissue mechanics in wound induced hair follicle regeneration of laboratory and spiny mice. Nat Commun 12: 2595. 10.1038/s41467-021-22822-9

Liechty K. W., Kim H. B., Adzick N. S., Crombleholme T. M. (2000). Fetal wound repair results in scar formation in interleukin-10–deficient mice in a syngeneic murine model of scarless fetal wound repair. J. Pediatr. Surg. 35 866–873. 10.1053/jpsu.2000.6868

Morioka N, Ganier C, Watt FM. Fetal Fibroblast Heterogeneity Defines Dermal Architecture during Human Embryonic Skin Development. J Invest Dermatol. 2025 May;145(5):1081-1091.e7. doi: 10.1016/j.jid.2024.12.027.

Morioka N, Ganier C, Watt FM. Fetal Fibroblast Heterogeneity Defines Dermal Architecture during Human Embryonic Skin Development. J Invest Dermatol. 2025 May;145(5):1081-1091.e7. doi: 10.1016/j.jid.2024.12.027

Peled ZM, Rhee SJ, Hsu M, Chang J, Krummel TM, Longaker MT. The ontogeny of scarless healing II: EGF and PDGF-B gene expression in fetal rat skin and fibroblasts as a function of gestational age. Ann Plast Surg. 2001 Oct;47(4):417-24. doi: 10.1097/00000637-200110000-00010.

Ramelet AA, Hirt-Burri N, Raffoul W, Scaletta C, Pioletti DP, Offord E, Mansourian R, Applegate LA. Chronic wound healing by fetal cell therapy may be explained by differential gene profiling observed in fetal versus old skin cells. Exp Gerontol. 2009 Mar;44(3):208-18. doi: 10.1016/j.exger.2008.11.004.

Ravanti L, Toriseva M, Penttinen R, Crombleholme T, Foschi M, Han J, Kähäri VM. Expression of human collagenase-3 (MMP-13) by fetal skin fibroblasts is induced by transforming growth factor beta via p38 mitogen-activated protein kinase. FASEB J. 2001 Apr;15(6):1098-100. doi.org/10.1096/fsb2fj000588fje

Scheid A, Wenger RH, Schäffer L, Camenisch I, Distler O, Ferenc A, Cristina H, Ryan HE, Johnson RS, Wagner KF, Stauffer UG, Bauer C, Gassmann M, Meuli M. Physiologically low oxygen concentrations in fetal skin regulate hypoxia-inducible factor 1 and transforming growth factor-beta3. FASEB J. 2002 Mar;16(3):411-3. doi: 10.1096/fj.01-0496fje.

Soo C, Hu FY, Zhang X, Wang Y, Beanes SR, Lorenz HP, Hedrick MH, Mackool RJ, Plaas A, Kim SJ, Longaker MT, Freymiller E, Ting K. Differential expression of fibromodulin, a transforming growth factor-beta modulator, in fetal skin development and scarless repair. Am J Pathol. 2000 Aug;157(2):423-33. doi: 10.1016/s0002-9440(10)64555-5.

Volk SW, Wang Y, Mauldin EA, Liechty KW, Adams SL. Diminished type III collagen promotes myofibroblast differentiation and increases scar deposition in cutaneous wound healing. Cells Tissues Organs. 2011;194(1):25-37. doi: 10.1159/000322399

Walraven M, Beelen RH, Ulrich MM. Transforming growth factor-β (TGF-β) signaling in healthy human fetal skin: a descriptive study. J Dermatol Sci. 2015 May;78(2):117-24. doi: 10.1016/j.jdermsci.2015.02.012.

Whitby DJ, Longaker MT, Harrison MR, Adzick NS, Ferguson MW. Rapid epithelialisation of fetal wounds is associated with the early deposition of tenascin. J Cell Sci. 1991 Jul;99 ( Pt 3):583-6. doi: 10.1242/jcs.99.3.583.

White P, Thomas DW, Fong S, Stelnicki E, Meijlink F, Largman C, Stephens P. Deletion of the homeobox gene PRX-2 affects fetal but not adult fibroblast wound healing responses. J Invest Dermatol. 2003 Jan;120(1):135-44. doi: 10.1046/j.1523-1747.2003.12015.x.
